# Bioconjugation Strategies for Microtoroidal Optical Resonators

**DOI:** 10.3390/s101009317

**Published:** 2010-10-18

**Authors:** Heather K. Hunt, Carol Soteropulos, Andrea M. Armani

**Affiliations:** 1 Mork Family Department of Chemical Engineering and Materials Science, University of Southern California, Los Angeles, CA 90089, USA; E-Mail: heather.hunt@usc.edu; 2 Department of Biomedical Engineering, University of Southern California, Los Angeles, CA 90089, USA; E-Mail: soteropu@usc.edu; 3 Ming Hsieh Department of Electrical Engineering-Electrophysics, University of Southern California, Los Angeles, CA 90089, USA

**Keywords:** bioconjugation, optical resonators, sensors, high quality factor

## Abstract

The development of label-free biosensors with high sensitivity and specificity is of significant interest for medical diagnostics and environmental monitoring, where rapid and real-time detection of antigens, bacteria, viruses, *etc.*, is necessary. Optical resonant devices, which have very high sensitivity resulting from their low optical loss, are uniquely suited to sensing applications. However, previous research efforts in this area have focused on the development of the sensor itself. While device sensitivity is an important feature of a sensor, specificity is an equally, if not more, important performance parameter. Therefore, it is crucial to develop a covalent surface functionalization process, which also maintains the device’s sensing capabilities or optical qualities. Here, we demonstrate a facile method to impart specificity to optical microcavities, without adversely impacting their optical performance. In this approach, we selectively functionalize the surface of the silica microtoroids with biotin, using amine-terminated silane coupling agents as linkers. The surface chemistry of these devices is demonstrated using X-ray photoelectron spectroscopy, and fluorescent and optical microscopy. The quality factors of the surface functionalized devices are also characterized to determine the impact of the chemistry methods on the device sensitivity. The resulting devices show uniform surface coverage, with no microstructural damage. This work represents one of the first examples of non-physisorption-based bioconjugation of microtoroidal optical resonators.

## 1. Introduction

The development of biosensors with high sensitivity and specificity is of significant interest to scientific communities, such as medical diagnostics and environmental monitoring, where rapid and real-time detection of antigens, bacteria, viruses, *etc.*, is necessary. A prime example for this need is the development of biosensors for detection in complex environments, such as whole blood or serum. Common, high sensitivity methods for detection in such complex environments include immunoassays, such as the ELISA assays and fluorescent immunoassays, which require the presence of a label moiety for detection [1–5]. However, these traditional, labeled sensors detect the presence of the label or probe rather than the molecule of interest, and require foreknowledge of the presence of the target. On the other hand, label-free sensors, such as electrical sensors [6,7], mechanical cantilever sensors [8–10], and optical sensors, such as optical waveguides [11–13], surface plasmon waveguides and resonators [14–19], and ring resonators [20], can detect the molecule of interest, but often have difficulty discriminating between targeted and non-targeted species in complex environments, such as serum and whole blood. Therefore, currently, traditional labeled biosensors have a significant advantage over label-free biosensors in many “real-world” applications.

This limitation of label-free biosensors can be eradicated by the addition of a component that adds specificity to the device. While the majority of previous research efforts on label-free biosensors have focused on the development of the sensor itself [6,10,21–30], specificity is an equally if not more important feature of any sensing platform, especially for detection in complex environments [31,32]. Label-free sensor performance can be improved by requiring the addition of a component, such as a probe molecule, that allows the sensor to selectively identify the target molecule [33,34]. In many sensing methods, this has been accomplished by surface immobilization of probe molecules via physical adsorption, self-assembly, or covalent attachment [35]. Of these techniques, covalent attachment provides the most stable immobilization towards changing environmental conditions, such as temperature, humidity, and pH. To date, the majority of research using optical sensors based on waveguides and surface plasmon resonance, have been paired through *physisorption* with enzymes [14,36], peptides [22,37,38], antibodies (or antibody fragments) [1,6,14,39,40], aptamers [41,42], and receptors [16,31,43–45] as environmental probes. However, recently, there has been increased interest in developing more robust surface functionalization protocols for optical devices, such as whispering gallery mode resonant cavities [46].

**Figure 1.**
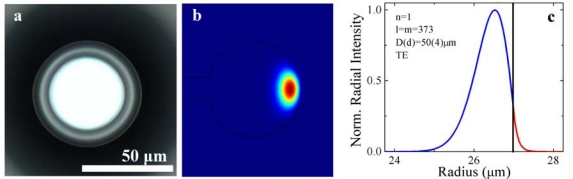
Optical resonant cavity. **(a)** An image of a microtoroid resonant cavity. **(b)** Finite element method simulation of the intensity of the optical field at 633 nm for a microtoroid cavity. As can be seen, the optical field is primarily confined inside the silica cavity, but a small portion evanesces into the environment. **(c)** The intensity profile of the optical field inside (blue) and outside (red) of the cavity. The black line indicates the air/silica interface. It can be clearly seen that the evanescent tail is approximately 100 nm.

Although initially designed for telecommunications, these whispering gallery mode microcavities have demonstrated unique capabilities in the biosensing arena primarily due to their very low optical loss, corresponding to very high sensitivities in biodetection [26,29,30,47–59]. Whispering gallery mode optical resonators efficiently confine light at specific resonant frequencies within the resonator periphery (Figure 1a). In these devices, the optical field is not completely confined to the resonator, but instead extends or evanesces into the surrounding environment, and interacts with its surroundings, thus enabling the detection and sensing capabilities of the resonators (Figure 1b). The primary gauge of resonator quality is the device’s quality factor, or Q factor, which describes the photon lifetime (τ_0_) in the cavity. For example, an ultra-high-Q device (Q > 100 million) has a photon lifetime greater than 100 ns. This long photon lifetime increases the interaction between the circulating photons and the environment, resulting in higher sensitivity as compared to more conventional methods.

While optical resonant cavities can be fabricated in many geometries, and from many different materials, the motivation to maximize the Q factor and the photon lifetime across a wide range of operating frequencies has led to silica-based, optical resonator devices that are circular in nature, such as microspheres, microrings, microdisks, and microtoroids [20,49,60,61]. The advantage of the latter three shapes is that they may be fabricated on a planar substrate via lithographic techniques, increasing ease of use and allowing potential integration with on-chip microfluidics. Of the planar microcavities mentioned above, the microtoroids have demonstrated the highest Q values (Q > 10^8^) in both water and in air [47,48,57,62,63].

Label-free whispering gallery mode optical resonators, especially those fabricated on a planar substrate, represent an intriguing platform for high sensitivity detection in complex environments. However, they must first be bioconjugated to add specificity to the device for optimal performance in these environments. Previous work on the bioconjugation of whispering gallery mode sensors focused primarily on resonant cavity detection, rather than the development of bioconjugation techniques, and did not study the effects of these techniques on the device sensitivity or the lifetime of the chemistry [64–66]. Therefore, it is crucial to develop and to fully characterize a covalent surface functionalization process which also maintains the optical device’s performance metrics. In the case of the whispering gallery mode sensor, the most important parameter is the Q factor of the cavity. Here, we demonstrate a facile method to impart specificity to optical microcavities without adversely impacting their optical performance (Q > 10^6^). Although our efforts have focused on the silica ultra-high-Q microtoroid microcavity, the techniques developed are transferable to other optical cavities, such as microrings, microspheres and microcylinders. This strategy could accelerate the development of label-free sensors for rapid diagnostics.

## 2. Experimental Procedures

Although ultra-high-Q optical resonators, such as microtoroids, have extremely high sensitivity, a measure of specificity must be imparted to the resonators in order to accurately detect specific interactions with the surrounding environment. Towards this end, the development of a library of surface modification techniques, which will enable specific sensing without deleterious effects on the device sensitivity, is of high importance to the field of biochemical sensing with label-free optical devices. Optimally, these surface modification techniques would result in an optical resonator whose surface is covered with one half of a binding pair (the probe molecule) that is capable of specific detection of a target molecule in a variety of environments, such as water, buffer, serum, *etc.*, with low rates of false positives. Silane-based chemistries, such as bifunctional silane coupling agents, which can react with a substrate on one end and organic functionalities on the other, promote the bonding of organic matter to an inorganic substrate [67]. These coupling agents are well-suited to the surface functionalization of silica-on-silicon optical resonators due to their incredible variety and commercial availability from companies such as Gelest and Dow Chemical. Additionally, their variety enables the ability to design an increasing number of bioconjugated surface complexes. However, these routes must be tailored to the resonant sensor, such that the surface functionalities have:

- low optical absorption at the wavelength of interest,
- length-scale compatible with the evanescent field,
- high density packing,
- specificity to only the target ligand,
- minimal reagent use, and
- high stability of the probe molecule to storage in air.

The first consideration for the attachment of molecular probes to the silica optical resonator via silane linker chemistry that must be addressed is the formation of a covalently attached, uniform monolayer of silanes on the resonator surface. This monolayer should be contained within the evanescent tail of the optical field, so the silane linker must be relatively short compared to typical high-molecular weight polymers. A uniform monolayer promotes a high binding efficiency of target molecules, and is less likely than a thick silane layer to negatively affect the performance of the resonator as a sensor. A second consideration is the preferential attachment of the probe molecules to the device and not to the surrounding chip. The third consideration, then, is the grafting of the probe molecules to the silanes’ functional groups, again with uniform coverage and high efficiency, resulting in a low number of unreacted organic functionalities in the silane layer. In the current work, we are interested in a high density packing of the probe molecules on the surface that can undergo binding with the target molecules of interest; in the future, the ability of this layer to prevent non-specific interactions with the device surface will be explored. Fourth, the number of reaction steps and reactants should be kept to a minimum to prevent device degradation through structural damage or surface defects. Therefore, our initial approach to imparting specificity to the optical resonators is based on grafting probe molecules to the surface of silica microstructures via bifunctional silane coupling agents. As a proof of concept, we investigate the biotinylation of ultra-high-Q microtoroids, and demonstrate the simplicity and suitability of this method for maintaining sensitivity of on-chip, silica optical resonators.

**Figure 2.**
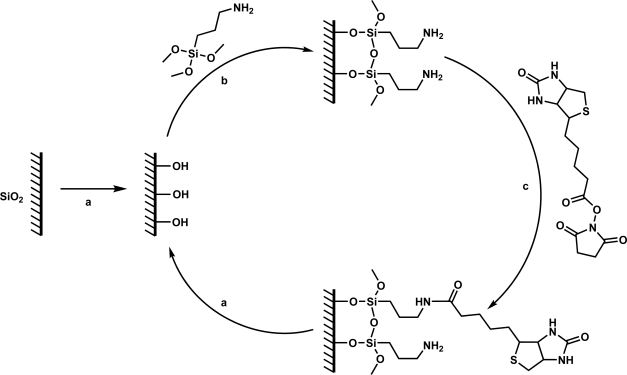
Overall reaction scheme for the biotinylation of silica microtoroids. **(a)** Hydroxylation of the silica surface. **(b)** Amination of the hydroxylated surface via silane coupling agent. **(c)** Biotinylation of the aminated surface via NHS ester chemistry.

The overall approach of our method is shown in Figure 2. In this approach, we selectively functionalize the surface of the silica microtoroids with amine-terminated silane coupling agents (approximately 9 Å at full extension) using two different methods: 1) organic solvent deposition and 2) vapor deposition grafting techniques [67]. The surface is then biotinylated via N-hydroxysuccinimide (NHS) ester chemistry at the amine end of the linker under typical literature conditions, using an NHS-biotin linker with a spacer arm of 13.5 Å [67]. NHS esters bind in an approximately quantitative fashion with primary amines, resulting in the formation of a stable amide bond. This reaction results in a biotinylated surface whose quality is highly dependent on the reaction conditions used. Given the length of the evanescent field from Figure 1c, at full extension, the biotin probe will be well within the evanescent tail of the optical field.

### 2.1. Device Fabrication

The silica microtoroids were fabricated using 2 μm thermal oxide on Si wafers (Montco Silicon, P-doped), following a well-established, three-step process: oxide patterning, silicon dry-etching, and laser reflowing [48]. First, the silica surface was lithographically patterned with 100 μm diameter circular pads using S1813 photoresist (Shipley). The oxide was then etched using Buffered Oxide Etch (Transene). Second, the silicon was isotropically dry-etched using a custom-built XeF_2_ etching system, forming a microdisk. Finally, the silica microdisks were reflowed to form toroids using a 50 W CO_2_ laser (Synrad).

### 2.2. Device Functionalization Protocols

All anhydrous solvents used during device functionalization were stored under UHP Ar. All chemicals and other solvents for the following reactions were used as received from their respective suppliers. The silica microtoroids fabricated following the above procedures were functionalized via three primary reaction steps: surface hydroxylation, amination, and finally, biotinylation. First, the silica surface was terminated with hydroxyl groups via typical literature procedures to promote covalent attachment of the silane coupling agent, using either O_2_ plasma treatment (120 W, 200 mTorr, 2 minutes) or a 70:30 H_2_SO_4_ (fuming, Aldrich): H_2_O_2_ (30 wt %, Aldrich) piranha etch for 45 minutes [68–70]. Following the piranha etch, the samples were cleaned with DDI H_2_O, and air-dried between 25–60°C for 30 minutes–24 hours to completely remove the water from the sample surface. Second, aminated silica surfaces were prepared by grafting silane coupling agents to the surface using two different methods: organic solvent deposition or chemical vapor deposition [41,67,71,72]. In the first method, a 2 mM solution of 3-aminopropyltrimethoxysilane (APTMS, Aldrich) was prepared in anhydrous toluene in a glove bag under a UHP Ar environment. The sample was then placed in the solution, and reacted at room temperature on an incubating rocker (VWR) for 10 minutes–4 hours to form a monolayer of silane on the surface. The sample was then removed from the reaction solution, and washed with toluene, ethanol, and then water for 5 minutes each on the incubating rocker. The sample was again air-dried between 25–60 °C for 30 minutes–24 hours to completely remove the water from the sample surface. In the second method (chemical vapor deposition), the hydroxylated sample was exposed to APTMS vapor in a vacuum desiccator under aspirator vacuum for 15 minutes–4 hours. Lastly, the aminated samples were then biotinylated via reaction in a 10 mM solution of NHS-Biotin (Pierce) in dimethylsulfoxide (DMSO, anhydrous, Aldrich) at room temperature for 30 minutes, on an incubating rocker. The surface was then washed with water and then acetone, as above, to remove any physically absorbed biotin from the surface, and air-dried at room temperature for 30 minutes.

Amine-terminated samples were fluorescently labeled using fluoroscein-5-isothiocyanate (FITC, Pierce), which forms a stable thiourea bond with the primary amines present on the surface in a 1:1 ratio. The FITC reaction solution was prepared in a darkened room by dissolving 1 mg FITC in 1 mL DMSO (anhydrous), and diluting it via addition of 50 μL of the solution to 1 mL of 0.1 M sodium carbonate buffer at pH 9.0 (VWR). The samples were gently inserted into this reaction solution in covered vials, placed in an ice bath, and reacted for at least 8 hours in an incubating rocker. Physically adsorbed FITC was removed via rinses with DMSO. The samples were then air-dried at 25–60 °C for 30 minutes–24 hours. Biotin-terminated samples were fluorescently labeled with Texas Red fluorescent dye conjugated to avidin protein (Invitrogen). Biotin-avidin binding was accomplished by reacting the biotinylated samples in a covered, 10 μg/mL solution of Texas Red-avidin protein conjugate in phosphate buffered saline (PBS, VWR) for 30 minutes at room temperature in an incubating rocker. The sample was removed from the solution and washed with PBS to remove physically-adsorbed reactant.

### 2.3. Device Characterization Protocols

The as-fabricated and surface-modified microtoroid devices were characterized qualitatively using a Nikon H550S optical microscope equipped with a Nikon digital camera, and NIS-Elements imaging software. The surface chemistry of these devices was explored via X-ray photoelectron spectroscopy (XPS, M-Probe ESCA) using a 1,487 eV Al Kα source, and a survey scan from 0 to 1,000 binding eV to identify all chemical species on the surface. Reaction efficacy and quality was monitored using fluorescent imaging with a Nikon ECLIPSE LV100D-U fluorescence microscope equipped with a Nikon digital camera at each reaction step. Intensity measurements were obtained using NIS-Elements imaging software and Texas Red and FITC excitation and emission filters, where appropriate. Lastly, ellipsometric measurements were taken using a Gaertner L166C ellipsometer to determine the film thickness of the initial silane layer. For the XPS and ellipsometry techniques, control samples consisting of surface-modified, non-patterned, silica-on-silicon wafers were used in place of the patterned samples.

**Figure 3.**
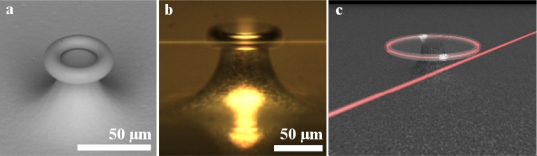
**(a)** Scanning electron micrograph of as-fabricated microtoroid. **(b)** Side view optical micrograph of microtoroid during device characterization. The tapered fiber can be clearly seen. **(c)** PovRay rendering of the microtoroid resonator and tapered optical fiber. The optical field is primarily confined inside the silica cavity, but a small portion evanesces into the environment.

The devices were characterized quantitatively by microcavity analysis at each reaction step to determine the impact of the surface functionalization methods on the device sensitivity and to evaluate the bioconjugation conditions best suited to ensuring the devices’ performance. The resonator quality factor was measured using a tapered optical fiber waveguide to couple power into the devices from a narrow linewidth, CW tunable diode laser centered at 635 nm (New Focus) (Figure 3b) [73–75]. The F-SV optical fiber (Newport) was tapered to ∼500 nm waist diameter by heating with an oxyhydric torch while stretching the fiber with a two-axis stage controller (Sigma Koi). During testing, the device was placed on a 3-axis nanopositioning stage (Optosigma), and the device was monitored using side- and top-view cameras simultaneously. Coupling into the resonator results in the excitation of the whispering gallery modes of the microcavity (Figure 3c). The resonance linewidth data was recorded using a digitizer/oscilloscope card which was directly integrated into the computer for automated data recording (NI, PCI-5114). The laser scan speed and scan direction was optimized to ensure that neither distorted the resonance lineshape. The quality factor of the microtoroid resonator was determined by calculating the resonance linewidth (full width at half-maximum) from the recorded spectra taken in the undercoupled regime [48,60,63,75–77].

## 3. Results and Discussion

Four different routes to biotinylation were explored for their suitability towards silica microtoroid resonators, based on combinations of hydroxylation (piranha etch, O_2_ plasma etch) and amination (organic solvent deposition, chemical vapor deposition) conditions. As previously mentioned, one of the key requirements for adding specificity included minimizing reagent use, the number of reaction steps needed, and harsh environmental conditions, which should minimize surface and structural damage. Therefore, the exploration of these four routes enabled the selection of a facile and efficient technique that can be applied to silica optical resonators without severely impacting device sensitivity.

### 3.1. Analysis of the Surface Functionalization

Our exploration of these specific combinations grew out of the requirements of grafting organosilanes to inorganic surfaces, such as silica, alumina, titania, *etc*. Typically, silane coupling agents (R(CH_2_)_n_SiX_3_, where R is an organic functional group and X is a hydrolyzable leaving group) undergo hydrolysis to labilize the leaving groups and form a reactive silanol intermediate species. This silanol species can couple to surface hydroxyl groups via condensation reactions and form stable siloxane bonds. However, the condensation reaction that couples the silane to the surface requires a high density of hydroxyl groups to form a uniform surface coverage [35,67,71,78]. Typically, the –OH surface density is increased by an oxidative piranha treatment, which leaves the surface extremely hydrophilic. This treatment, however, may be too harsh in terms of temperature and acid strength with regard to microstructured optical devices, and may lead to difficulties in preserving the structural and surface integrity of the devices. An alternative is the use of O_2_ plasma, which is typically used in fabrication processes to clean photoresist from structured surfaces. O_2_ plasma etching is simple, environmentally benign, minimizes device handling, and does not require post-treatment to remove excess water from the surface. On the other hand, O_2_ plasma treatment may not yield as high of a surface hydroxyl density, leading to low surface coverage of the organosilane. Therefore, it is important to identify which route is most compatible with our devices.

After hydroxylation, the silane coupling agent can be grafted to the surface. Here, we used APTMS as our silane coupling agent due to its short organic tether between the silica head and the terminal amine, which should ensure that the final biotin probe is well within the evanescent tail of the functioning device. Additionally, the amine portion should not cause additional material losses due to absorption at the wavelength of interest. Surface grafting of APTMS may be accomplished via reaction in aqueous or organic solvent, or through vapor phase deposition. Typically, alkoxysilanes (where X is an alkoxy group) are used to functionalize the surface of microstructured devices, since they minimize the formation of a silica polymer network on the surface and help achieve monolayer coverage, due to the lower hydrolyzability of the alkoxy leaving groups [67]. This lower hydrolyzability can lead to the necessity of hydrolyzing the alkoxysilane in water first, as they are unreactive to surface hydroxyls at ambient conditions in their native form. Unfortunately, aqueous solvent deposition causes the same multi-layer deposition problem as working with non-alkoxysilanes. Fortunately, chloro- and methoxysilanes, such as APTMS, do not need pre-hydrolysis, and can be coupled to the substrate in a uniform monolayer through siloxane bonding via anhydrous organic solvent deposition or vapor deposition [67]. In both deposition cases, since water is not present in the system, the three-dimensional silica polymer network does not form. Either route may be appropriate for microstructured optical devices, but vapor deposition typically requires less chemical use, post-treatment, and handling than organic solvent deposition. Here, we investigate both routes, along with the hydroxylation alternatives, to determine their impact on the devices.

**Figure 4.**
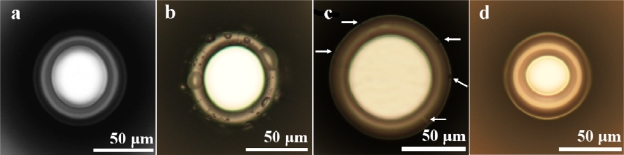
Optical micrographs of silica microtoroids. **(a)** As-fabricated microtoroid. **(b)** Microtoroid after piranha etch. **(c)** Microtoroid after piranha etch and 24 h drying at 100 °C. The white circles indicate regions of structural damage. **(d)** Microtoroid after O_2_ plasma treatment.

Figure 4, below, shows optical micrographs of typical silica microtoroids before and after hydroxylation using either piranha or O_2_ plasma treatment. The primary issue with piranha treatment is that it is carried out in an aqueous environment, and upon reaction completion and device removal from the solution, water must be removed from the now hydrophilic surface. Typically, complete removal is not possible via air-drying at room temperature. Additionally, drying with a heat gun or air nozzle results in fractured toroids. To overcome this, the device-laden substrate may be placed on a hot plate, or in an oven, and the temperature raised to 35 °C. Temperatures above this result in structural damage to the toroid itself (as indicated by the white circles in Figure 4c). Typically, this damage presents itself either as thin cracks radiating outward from the pillar (thin white lines in the image), or small divots (black spots in the image) in the surface of the toroid. Either type of damage will reduce the performance of the device. In comparison, O_2_ plasma treatment requires minimal device handling and no post-treatment, and does not result in surface- or structurally-damaged devices. Additionally, the time requirements for O_2_ plasma treatment are significantly less than those for piranha treatment.

**Figure 5.**
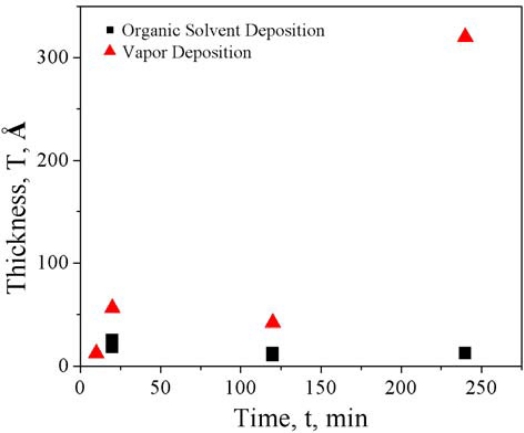
Ellipsometry data showing film thicknesses after various reaction times for organic solvent deposition and vapor deposition.

Figure 5 shows the ellipsometry measurement of the film thickness of the silane film resulting from amination of the surface of control wafers that had been hydroxylated via O_2_ plasma treatment over various reaction times. It is interesting to note that while organic solvent deposition resulted in an approximately constant film thickness over time, increasing the time of vapor deposition resulted in non-uniform film thicknesses, increasing in an approximately exponential trend. This behavior is typical for chemical vapor deposition processes [79]. The difference in these deposition rates is due to the difference in film deposition mechanisms between chemical vapor deposition and organic solvent deposition of alkoxysilanes [80]. We refer the interested reader to Duchet *et al.* for a discussion of these mechanisms [80]. From this data, it is apparent that 10 minutes of chemical vapor deposition is sufficient to create a uniform monolayer, approximately 8–10 Å thick.

**Figure 6.**
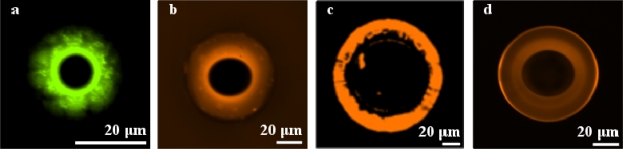
Fluorescent micrographs of microtoroids after labeling. **(a)** FITC-labeled, aminated microtoroid after piranha treatment, followed by organic solvent deposition. **(b)** Texas Red-labeled, biotinylated microtoroid after piranha treatment, followed by vapor deposition. **(c)** Texas Red-labeled, biotinylated microtoroid after O_2_ plasma treatment, followed by organic solvent deposition. **(d)** Texas Red-labeled, biotinylated microtoroid after O_2_ plasma treatment, followed by vapor deposition.

Figure 6 shows fluorescent micrographs of FITC- and Texas Red-labeled, aminated and biotinylated (respectively) toroids obtained using the four combinations of hydroxylation/amination procedures. The combination of O_2_ plasma treatment and vapor deposition results in the most uniform surface coverage of the toroids. Interestingly, in each case, amination occurred only on the silica toroid itself, and not on the silicon wafer. This was interesting to us, as the process of hydroxylating the surface of the device and the substrate should lead to the ability to aminate both surfaces (the silica of the device as well as the silicon of the substrate). As an exploration of why this preferential amination appeared only on the silica, we hydroxylated (using O_2_ plasma) and aminated three different sets of planar substrates: a bare silicon wafer, a bare silicon wafer subjected to XeF_2_ etching using the same parameters as used to make the devices, and a 2 μm SiO_2_ on silica wafer. The aminated samples were subjected to FITC labeling, and the average intensities of the resulting surfaces were measured, normalized by the background intensity of each sample. Interestingly, the average intensity (a.u.) of the FITC labeled surfaces showed that XeF_2_ etching seems to reduce the overall amination of the silicon surface (intensity of the bare wafer: 1161, intensity of the bare wafer subjected to XeF_2_ etching prior to functionalization: 364, and intensity of the thermal oxide wafer: 924). Clearly, the exposure to the XeF_2_ is inducing the improved functionalization of the device surface. However, at this point, the precise mechanism is unclear.

The combination of piranha treatment and organic solvent deposition typically led to the poorest results, with severe surface clumping, indicating either a non-uniform coverage by the silane coupling agent, or the presence of water drops remaining on the surface after hydroxylation. Clumping of the silane layer during organic solvent deposition could be minimized by carrying out the reaction on a rocker; however, it was never entirely prevented, as shown by Figure 6c, which shows the O_2_ plasma treatment/organic solvent deposition combination. Note that the image was deliberately overexposed to help show the detail of surface clumping and surface damage from organic solvent deposition.

The results from the series of experiments are summarized in Table 1. Overall, the difficulties involved with proper drying of the piranha-treated samples and with clumping of the organic solvent deposition samples indicated that these methods were inappropriate for further use. Note that, due to its poor performance, the piranha treatment/organic solvent deposition combination was not pursued to the point of biotinylation. Additionally, several biotinylated samples were labeled with FITC to determine the extent of the NHS ester reaction with surface amine groups; fluorescent microscopy did not yield samples with intensities above the substrate background (no fluorescent labeling occurred), indicating that the density of amines remaining after biotinylation was too low to be detected by fluorescent imaging techniques. This implies that the reaction biotinylated all the amine groups present or that steric hindrance prevented access of the FITC to the remaining amines on the surface. Regardless, the inaccessibility of the amine groups for reaction with FITC, a relatively small molecule compared to antibodies and proteins, indicates that the presence of the amine groups should lead to minimal activity towards non-targeted species in the environment.

**Figure 7.**
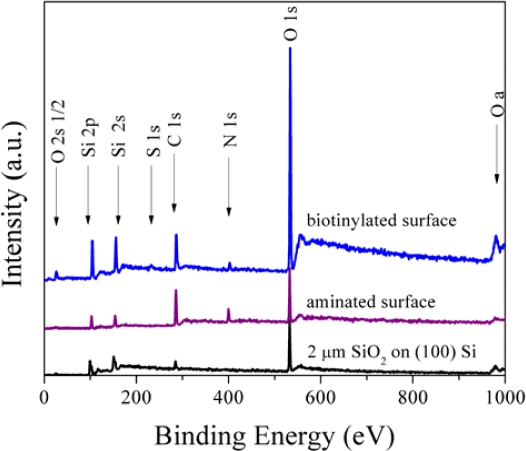
Chemical composition of control surfaces after each reaction step (using O_2_ plasma etching and vapor deposition conditions).

Although fluorescent labeling provides confirmation of the presence and uniformity of the surface species, it is an indirect method to do so. An additional confirmation may be obtained using X-ray photoelectron spectroscopy (XPS), which directly probes the surface composition through irradiation of the sample with X-rays, resulting in the ejection of electrons from the top ∼10 nm of the sample. Simultaneously measuring the kinetic energy and number of electrons ejected allows us to quantify the elemental source of these electrons. Figure 7 show the resulting surface composition at each reaction step obtained through XPS data collection. The C (1s) signal that appears in the spectra is from primarily adventitious carbon in the ultra-high vacuum chamber, so direct comparison in terms of intensity of these peaks between runs is inappropriate, as the signal from the sample is masked by varying amounts of carbon in the chamber. Additionally, it is difficult to directly compare the intensities of the peaks between runs, since slight differences in spectrometer environment can lead to significant differences in peak intensities between runs (for instance, the Si (2p) and Si (2s) signals in each spectra); hence the designation of arbitrary units for the intensity data. Overall, the XPS data shown provide an additional confirmation of the success of each reaction step via the introduction of nitrogen (N) and sulfur (S) peaks into the spectra after amination and biotinylation, respectively. The combination of fluorescence imaging and XPS verify both the surface chemistry and surface quality of the samples.

### 3.2. Stability of the Surface Chemistry

**Figure 8.**
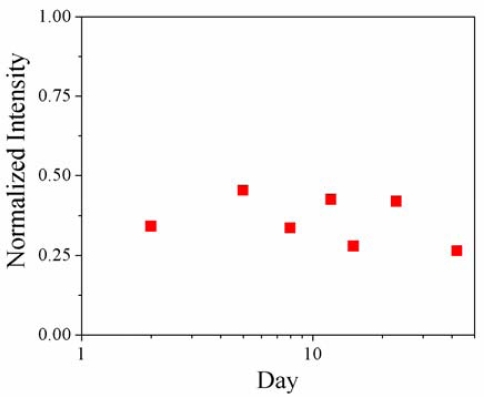
Fluorescent labeling intensities of biotinylated devices for given storage times, normalized according to device surface area. Note that the x-axis is plotted on a log-scale.

To determine the stability of the biotinylated devices for long-term storage in ambient conditions, seven chips, each containing 1–5 toroids, were biotinylated at the same time (day 1), then stored for various time-frames, bound with an avidin-Texas Red complex, and subjected to fluorescent microscopy. The intensity due to the binding reaction of biotin and avidin, and how it changes over storage time, can be used as a measure of the degradation or instability of the biotin probe with respect to avidin over time. Figure 8 shows the average device intensity, normalized by toroid surface area, over approximately 1.5 months of storage. It is important to note that long-term storage does not have a significant negative impact on the stability of the probe molecules attached to the device in terms of its ability to bind with avidin, as the normalized intensity holds steady around a normalized intensity of 0.35. Therefore, these devices could be fabricated and bioconjugated in advance, and stored until they were needed for use.

### 3.3. Quality Factor Study

**Figure 9.**
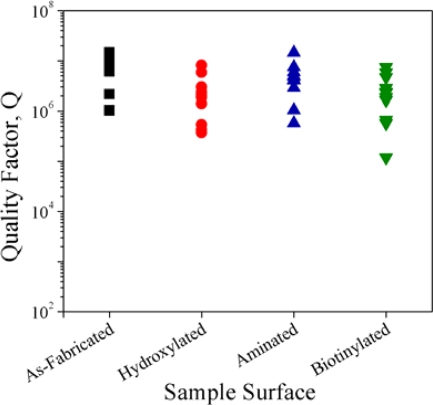
Effect of surface functionalization on the quality factor of the microtoroids at each reaction step.

**Figure 10.**
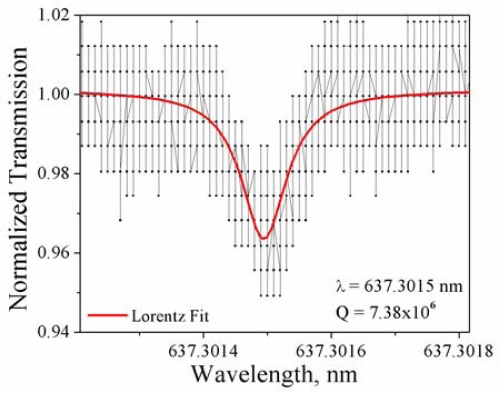
Transmission spectra of biotinylated toroid, showing a single, high-Q resonance (data connected by a solid black line) with the corresponding Lorentz fit (solid red line)

The as-fabricated and functionalized microtoroids were tested at each reaction step to determine the effects of functionalization on the sensitivity of the device at 633 nm. This wavelength was chosen based on the typical sensing environment (buffer or serum) in which these devices would be used [81]. Absorption of water in the near-IR to IR is very high (for example, at 1540 nm the loss is 11.8 cm^−1^) [82], so device sensitivity would be limited in this regime. However, at shorter wavelengths, absorption is reduced (for example, at 600 and 625 nm, the absorption of water is 0.002 and 0.003 cm^−1^, respectively) [82]. Therefore, the 635 nm wavelength was selected for this study. Figure 9 shows the results of a quality factor study of the bioconjugated devices after each reaction step. It is interesting to note that hydroxylation leads to a slight overall decrease in Q-value; however this is recovered after amination, indicating the formation of siloxane bonds and reduction of –OH density, which has a higher absorbance at 635 nm than the siloxane groups [78]. In this case, we require the maintenance of the Q above 10^6^ after biotinylation for these devices to be used as sensors in aqueous environments, for such applications as medical diagnostics or environmental monitoring. As Figure 9 shows, 8 of the 11 biotinylated microtoroids showed resonances corresponding to Q-values above 10^6^. A typical transmission spectra of the final, biotinylated toroids is shown in Figure 10. This Q-study demonstrates the effectiveness of our bioconjugation method, and shows that this method does not adversely impact the sensitivity (Q-factor) of the devices.

## 4. Conclusion and Future Outlook

Silica microtoroidal optical resonators were successfully conjugated with biotin, without surface or structural damage, and without a significant, negative impact on the overall sensitivity of the devices. The most successful bioconjugation protocol examined was the covalent attachment of biotin to the silica surface through a silane coupling agent via O_2_ plasma treatment, followed by vapor deposition of the silane. This strategy is a simple method for imparting specificity to highly sensitive optical devices, and could easily be extended to other sensor structures due to its flexibility and low impact on the device. This method opens the possibility for a reagentless “recycling” of functionalized devices by the removal of the organic surface coverage via O_2_ plasma etching after the sensor has been used. The covalent nature of attachment presents a significant improvement in terms of environmental stability compared with other methods of device functionalization, such as physical adsorption. The method described here results in devices whose probe molecules are stable for long-term storage. Additionally, this technique allows for the controlled presentation of the probe to the surrounding environment, and avoids background binding on the substrate due to the non-biological nature of the reaction strategy and the functionalization of the toroid surface only. Lastly, this method improves the performance of optical devices used as sensors by increasing the specificity to a target of interest.

This work represents one of the first examples non-physisorption-based bioconjugation of optical microtoroid resonators that can be used for the label-free detection of biomolecules. The primary advantage of a device that has been functionalized using the methods described above is that a wide array of biomolecules can be studied, since the functionalization process does not limit the study to a specific ligand-substrate pair, or even a specific type of molecule. For example, the protocols developed here are easily extended to a wide variety of probe molecule/target molecule pairings by using the high affinity of biotin for (strept)avidin-modified probes molecules, following the Avidin-Biotin Complexation (ABC) technique [83], allowing the easy interchange of the terminal functional group probes on the surface of the devices. Lastly, the protocols demonstrated here will result in new methods to produce highly sensitive and highly specific sensor devices that can be used for other applications, such as rapid medical diagnostics and environmental monitoring [1,31,32,84–86].

## Figures and Tables

**Table 1.** Summary of the effects of hydroxylation and amination surface functionalization techniques on microtoroid surfaces.

	**O_2_Plasma Etch**	**Piranha Etch**
**Organic Solvent Deposition**	Uneven coating of amine layer No structural damage	Water droplets on surface Structural damage from high temperatures used to remove accumulated surface water Clumping of amine layer
**Vapor Deposition**	No structural damage No water droplets on surface Uniform coating of amine layer No clumping visible	Water droplets on surface Structural damage from high temperatures used to remove accumulated surface water Clumping present to a lesser degree than organic solvent deposition
